# Endoscopic Carpal Tunnel Release using a modified application technique of local anesthesia: safety and effectiveness

**DOI:** 10.1186/1749-7221-3-11

**Published:** 2008-04-25

**Authors:** Abdullah Nabhan, Basem Ishak, Jehad Al-Khayat, Wolf-Ingo Steudel

**Affiliations:** 1 Department of Neurosurgery, Neurosurgical Department, University of Saarland, Homburg, Germany; 2 Department of Anesthesia, Anesthesia Department, University of Saarland, Homburg, Germany

## Abstract

**Background:**

Local anesthesia is widely used for open carpal tunnel release. However, injection of local anesthesia as described by Altissimi and Mancini (1988) can interfere with endoscopic carpal tunnel release, by increasing the bulk of synovial layers and consequently result in worsening of the view.

**Purpose:**

The purpose of this study was to evaluate the safety, efficacy using modified technique for application of local anesthesia.

**Methods:**

33 patients suffering from gradual increasing symptoms of carpal tunnel syndrome. The patients were also asked to evaluate the pain associated with injection as well as tourniquet during surgery using Visual Analogue Scale (VAS) (ranging from 0 = no pain to 10 = maximum pain).

**Results:**

One patient required additionally local anesthesia because of mild pain in the hand. The tourniquet was inflated for 13.00 (2.8 min). The pain score related to injection was 2.5 (0.8) and to tourniquet was 3.6 (0.9). Inflation of the tourniquet was well tolerated by all patients. Postoperative neurological sensory and motor deficits related to surgery and local blocks were not occurred.

**Conclusion:**

Endoscopic release of the carpal tunnel syndrome in local anesthesia is effective, well tolerated and safe. This kind of application of local anesthesia did not reduce visibility.

## Introduction

Anesthetic options for Endoscopic Carpal Tunnel Release (ECTR) include: general anesthesia (GA), local anesthetic (LA) infiltration, intravenous regional anesthesia (IVRA), and peripheral nerve blocks (PNB). Peripheral nerve blocks can be done either proximally at the brachial plexus or more distally at the peripheral nerves. The surgery is usually done either in wrist block or within intravenous application of local anesthesia [1]. For these ambulant procedures, it seems that local anesthesia should be especially favoured. Such a local infiltration is easy and quick to perform, especially the subcutaneous application. However, local Anesthesia has the reputation of being of limited interest because they may cause anatomical distortion at the site of incision [2].

Alternatives to local anesthesia, such as brachial block or IVRA, are more time consuming and the presence of an anesthetist has been recommended when these techniques are used [3].

This study was designed to verify the efficacy, tourniquet and injection associated pain with LA.

## Methods

This study was approved by the local ethical committee of Saarland (Germany).

33 patients with evidence of carpal tunnel syndrome were admitted to our neurosurgical department. After evaluation of the diagnosis, patients were informed about the study and invited to participate in the study. Medical history and physical examination, as well as Nerve conduction velocity (distal motor latency, dml) were obtained preoperatively. Inclusion, exclusion criteria are listed in table 1. All patients were enrolled in this study having giving written informed consent after being informed of the purpose of the study. The surgery was performed from one surgeon from the panel of authors (A. N.). All patients had an endoscopic carpal tunnel release (ECTR) of the median nerve in local anesthesia (LA) (Prilocain).

**Table 1:** Inclusion/exclusion criteria

Inclusion criteria	Exclusion criteria
Age rang 18 – 70 years	Prior surgery at the wrist
Both genders	Allergic to prilocain
Hand or wrist pain with paresthesias or numbness in the first three or all fingers	Deformity of the wrist bone
Symptoms presents for at least three months	Pregnancy
No prior surgery at the wrist	Coagulopathy
Willing and able to provide informed consent ability to take part on the study	
Median nerve distal motor latency > 4,5 ms	

The patients were also asked to evaluate the pain associated with injection as well as tourniquet during surgery using Visual Analogue Scale (VAS) (ranging from 0 = no pain to 10 = maximum pain).

Follow-up examinations were done pre- and postoperatively. Data are presented as mean value and Standard deviation.

### Technique

**Figure 1:**
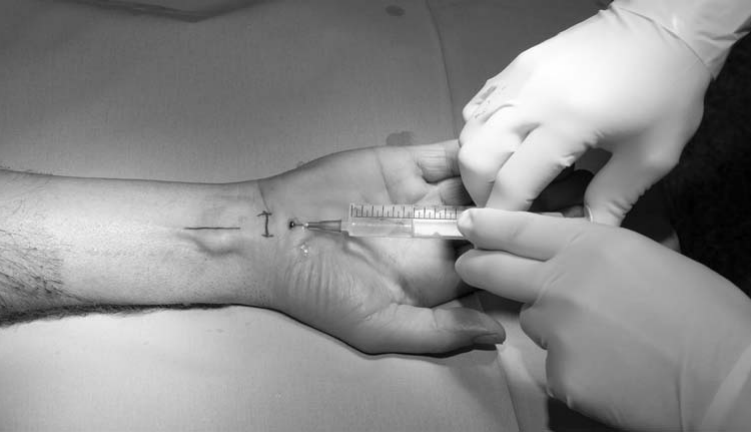
Subcutaneous infiltration of 10 ml 1% prilocain with a common 22G-Syringe-Needel in the palmar proximal wrist to block the median nerve.

**Figure 2:**
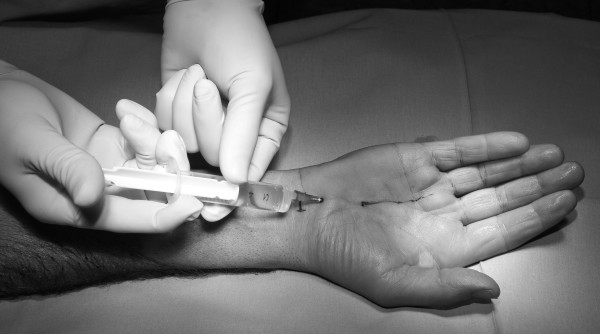
A second injection of 10 ml 1% prilocain subcutaneous at the volar side of the hand was done.

The median nerve passes between the flexor carpi radialis tendon and the Palmaris longus tendon. After positioning the arm, the area of in skin incision directly ulnar to the palmaris longus tendon was marked with a water resistant pen (Figure 1). Subcutaneous application of 10 ml Prilocain with a common 22G-syringe needle in the palmar proximal wrist as shown in Figure 1. Then the needle should be positioned to the distal wrist down to the palm and should not penetrate the ligamentum carpi transversum. At this stage the application of 10 ml LA was made (Figure 2). The infiltrated area was smoothly kneaded with few gauze compresses. In order to keep the tourniquet time as short as possible, a sterile elastic bandage is used to exsanguinate the arm and the tourniquet is inflated just before starting the operation. The effectiveness of the sensory block was evaluated with a needle at in the median nerve territory.

### Operative procedure

**Figure 3:**
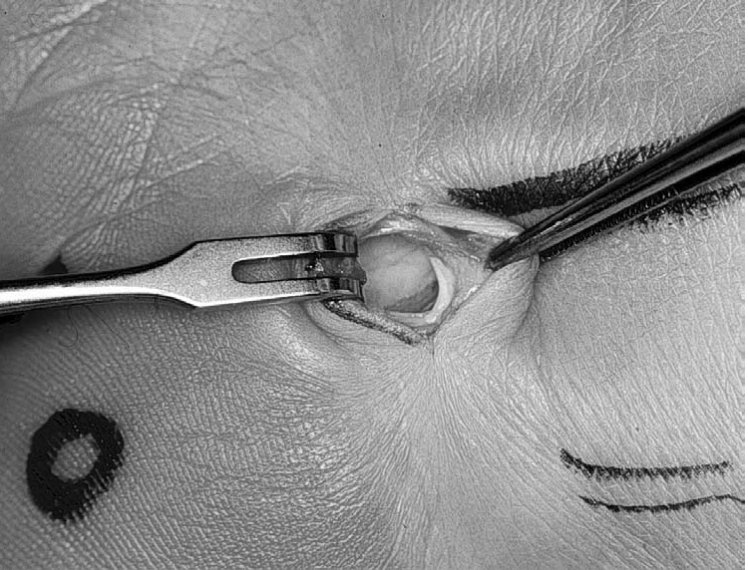
Incision and exposure of the median nerve.

After disinfection of the operation field, a transverse skin incision of circa 1.5 cm directly ulnar to the palmaris longus tendon was made. The palmar aponeurosis was divided and the median nerve identified (Fig. 3). Directly ulnar to the median nerve, the carpal canal was entered with a synovia elevator followed by a dissector to mobilize the nerve. After the removal of the dissector, the endoscope was inserted in the direction of the fourth finger until the distal end of the carpal ligament could be seen. The endoscope was The MicroAire Carpal Tunnel Release System (MicroAire Surgical Instruments LLC, Edlich Drive, Charlottesville). The carpal ligament was then divided from distal to proximal end of the ligament. The endoscope was reinserted to confirm complete division of the ligament. Skin was sutured with 4/0 interrupted stitches. The tourniquet was released and external compression exerted for 5 minutes to reduce the postoperative bleeding risk.

## Results

**Figure 4:**
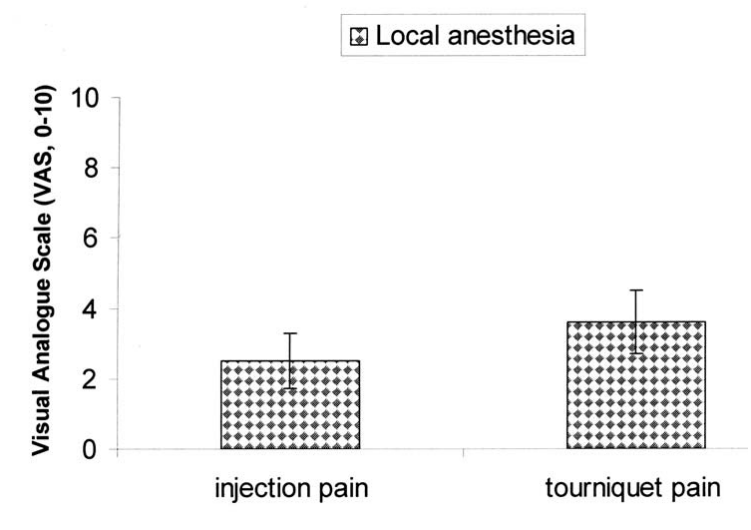
**Mean value and standard deviation of injection and tourniquet pain with the Visual Analogue Scale (0–10).** Pain ranging from 0 = no pain, 10 = unbearable pain.

One patient required additionally local anesthesia because of mild pain in the hand. The tourniquet was inflated for 13.00 (2.8 min). The pain score related to injection was 2.5 (0.8) and to tourniquet was 3.6 (0.9) (Figure 4). Inflation of the tourniquet was well tolerated by all patients. Postoperative neurological sensory and motor deficits related to surgery and local blocks did not occur.

## Discussion

There are various techniques of local anesthesia described by Altissami and Mancini [4], Wood and Logan [5] and Gale DW [6].

Altissimi and Mancini [4] injected local anesthetic around the median nerve under the flexor retinaculum for open carpal tunnel release. This technique potentially reduces visibility, and thus has a significant risk of nerve injury [5]. Wood and Logan 1999 [5] gave a subcutaneous injection of local anesthetic to allow a proximal wrist incision and an intraoperative injection of local anesthetic using a catheter with a blunt ended trochar. This method is time consuming, especially in view of tourniquet pain. Moreover deflation and a new inflation trial are not possible in cases of venous congestion.

Patil [7] compared two techniques of local anesthesia (the Gale technique 1990 and the Altissimi and Mancini technique 1988). Both were described for open carpal tunnel release.

This study did not support the concept that performing LA increases risk for nerve damage or causes anatomical distortion [2,4]. The risk of transient or permanent nerve damage following blocks, which is estimated to be between 2.1 and 9%, was thought to be caused by the deep injection of anesthesia [8,9].

Most investigators of endoscopic carpal tunnel release are skeptical about using local anesthesia [4,10]. They believe that the tourniquet pain increases with the use of local anesthesia. Since a tourniquet has to be used, the operation must be short and the tourniquet time should not exceed 20 to 25 minutes [11]. Moreover the application of local anesthesia as we described does not cause nerve damage. In the current study, the application of subcutaneous local anesthesia for endoscopic release of the carpal tunnel was effective. Furthermore, it is less invasive and simpler in comparison to surgery with other anesthetic options for ECTR, such as general anesthesia, intravenous regional anesthesia or peripheral nerve blocks. It's practical and can be done by the surgeon himself.

Another point of interest is that, in cases of venous congestion, deflation and re-inflation is only possible using local anesthesia.

No complaints of paresthesia were seen during injections of local anesthesia. Injection-associated problems such as increased thickness of the synovial layer or decreased endoscopic view did not occur. No instances of tendon or nerve injury or hematoma were seen.

Endoscopic release of carpal tunnel ligament in local anesthesia is safe and effective. It is less invasive and cost-saving.

## Competing interests

The authors declare that they have no competing interests.
